# As Evidence-Based Tumorigenic Role of Epstein-Barr Virus miR-BART1-3p in Neurological Tumors

**DOI:** 10.31557/APJCP.2021.22.1.257

**Published:** 2021-01

**Authors:** Mohammad Karimzadeh, Alireza Tabibzadeh, Mohsen Moghoofei, Saeedeh Abbasi, Javid Sadri Nahand, Farzin Sadeghi, Mohammad Hadi Karbalaie Niya, Hossein Keyvani, Farah Bokharaei-Salim, Moein Esghaei, Maryam Esghaei

**Affiliations:** 1 *Department of Virology, Faculty of Medicine, Iran University of Medical Sciences, Tehran, Iran. *; 2 *Department of Microbiology, Faculty of Medicine, Kermanshah University of Medical Sciences, Kermanshah, Iran. *; 3 *Gastrointestinal and Liver Disease Research Center (GILDRC), Iran University of Medical Sciences, Tehran, Iran. *; 4 *Laboratory of National Center, Vice Chancellor for Health, Iran University of Medical Sciences, Tehran, Iran. *; 5 *Cognitive Neuroscience Laboratory, German Primate Center - Leibniz Institute for Primate Research, Göttingen, Germany. *

**Keywords:** Neurological tumors, Epstein, barr virus, miR-BART, microRNA

## Abstract

**Introduction:**

Central nervous system tumors are a diverse group of tumors that account for 2% of all adult cancers and 17% of childhood malignancies. Several internal and external risk factors are involved in the development of this cancer such as viral infections. The aim of this study was to the determination of the EBV infection frequency and the expression level of *miR-122* and *miR-BART* in CNS tumors samples.

**Methods:**

One hundred and thirty-eight fresh tissue sample (106 case and 32 control) was collected from CNS specimens. The presence of Epstein-Barr virus (EBV) DNA was examined by PCR assay and the expression level of *miR-122 *and* miR-BART* were evaluated by using real-time PCR assay in CNS tissue samples.

**Results:**

EBV DNA was detected in 17% (18 of 106) of tumors tissue samples and 6.4% (2 of 32) of control samples. according to results, there was a significant relationship between the presence of EBV-DNA with CNS tumors. Additionally, the expression level of *miR-122* was significantly downregulated in the EBV-positive sample compared to that of the EBV-negative sample. Also, the level of *EBV-BART1-3p* expression was significantly higher in EBV-positive tumors samples than EBV-positive normal samples.

**Conclusion:**

The results of this study suggest that the EBV could change the condition of cancer cells by altering the expression of *miR-122 *and *EBV-BART1-3p* and maybe contribute to the development of cancer cells. However, the role of viral infections in CNS cancer requires further studies.

## Introduction

One of the most important malignancies in human is the central nervous system (CNS) tumors that constitute approximately 2% of all human adulthood cancers (Sadeghi et al., 2015a; He et al., 2016). Among the various types of CNS tumors including glioma, meningioma, and ependymoma arising from brain glial cells, meninges, and spinal cord, meningioma and glioblastoma are the most common type of these cancers (He et al., 2016; Sadeghi et al., 2015b; Xing et al., 2016; Ohgaki and Kleihues, 2005). Although many studies have been done for detection of different tumors in CNS, the pathogenesis and etiology of many CNS tumors are still understood (Akhtar et al., 2018a; Kofman et al., 2011). It has been reported that some of the risk factors including ionizing radiation, radiation of cellular phones, electromagnetic fields, head injury, genetic polymorphisms, neuro-carcinogenic chemicals, atopic diseases and infectious disease (e.g. viral infections) play a critical role in the development of CNS tumors (Strojnik et al., 2017; Andrei et al., 2019; Khodabandehlou et al., 2019). In addition, several studies demonstrated which the genome of some viruses, such as Epstein-Barr virus (EBV), Human Cytomegalovirus, varicella-zoster virus, Human herpesvirus 6, herpes simplex virus type 1 and herpes simplex virus type 2, BK virus, Parvovirus B19 John Cunningham virus, and simian vacuolating virus 40 have been isolated frequently from CNS tumors samples (Bhattacharjee et al., 2018; Yi et al., 2009; Amon and Farrell, 2005; Etemadi et al., 2017). 

EBV is a common human, double-stranded DNA virus belonging to the Herpesviridae family. EBV is one of the most frequently contracted herpesviruses that about 95% of the adult human population infected. In 1964, EBV was identified as the first human oncovirus (Mui et al., 2019). Primary EBV infection commonly events during childhood and is generally asymptomatic. However, EBV infection in young adulthood and adolescence caused infectious mononucleosis (IM) in 35-50% of the cases, and similar to other herpesviruses, following primary infection, EBV establishes a lifelong latency. EBV is the causative factor of infectious mononucleosis and is associated with the B-cell malignancies development, including Burkitt’s lymphoma, Hodgkin’s lymphoma, non-Hodgkin’s lymphomas, central nervous system lymphomas, AIDS-associated lymphoma, post-transplant lymphoproliferative disorder (PTLD), as well as T-Cell and NK-Cell Lymphomas (Moghoofei et al., 2019). 

MicroRNAs (miRNAs) are small non-coding RNAs (18–23 nucleotides) that recognize specific target mRNA sequences within the 3′ untranslated region and repress targeted gene expression through inducing mRNA degradation or translational suppression (Moghoofei et al., 2018, Sadri Nahand et al., 2019). Accumulating evidence has demonstrated that human miRNAs play an important role in several diseases such as viral infection disease and cancer (Rao et al., 2017). MiR-122 is the liver-specific miRNA and has a regulator role in liver development and in liver diseases. Furthermore, *miR-122 *operates as a tumors inhibitors gene or a deregulated factor in different types of malignancies including bladder, liver, gastric, and breast cancers (Wang et al., 2016, Sadri Nahand et al., 2019, Bandiera et al., 2015, Wang et al., 2014, Plaisance-Bonstaff and Renne, 2011, Zhang et al., 2018, Klinke et al., 2014). In addition to eukaryotes, miRNAs are also expressed in some of the viruses (EBV, HCMV, KSHV, HIV) (Cai et al., 2015). EBV encodes 44 mature miRNAs and 25 miRNA precursors from two regions of the genome: BamHI fragment A rightward transcript (BART) and BamHI fragment H rightward reading frame1 (BHRF1) (Strojnik et al., 2017). Noteworthy, previous researchers have shown that BART cluster miRNAs are effective in the process of tumors initiation (Leibovitch et al., 2016). It has been reported to EBV miRNAs play a critical role in immune evasion, latency maintenance, progression and development of the EBV-associated tumors through regulating both human and viral gene expression (Fonseca et al., 2015). Although in some studies EBV-DNA has been detected from glioblastoma and astrocytoma (Ai et al., 2012; Leibovitch et al., 2016; Fonseca et al., 2015; Zavala-Vega et al., 2017a), the expression pattern of EBV miRNAs is not well evaluated yet. The aim of this study is to investigate the presence of the EBV and evaluating the expression level of miR-122 and miR-BART1-3p in CNS tumors specimens.

## Materials and Methods

*Study population*

From January 2016 to November 2019, one hundred and three CNS tumors biopsy and blood samples were collected from patients who underwent surgical operations at The Neurosurgery Department of Rasoul-Akram Hospital in Tehran, Iran. It should be noted that samples were obtained before chemotherapy. Furthermore, thirty-two CNS samples, as the control group, were collected from autopsy tissue samples, that were not related to the neurological disorders as a cause of death Table 2. The CNS tumors type was conﬁrmed as part of the routine histopathological examination in the Pathology Department of Rasoul Akram Hospital. Case and control specimens were matched on sex and age. Before surgery operation time, except for one patient, all patients had a white blood cell count above 4,500 cells per microliter. Besides, none of the individuals received immunosuppressive drugs other than dexamethasone of 8 mg/m^2^, which was prescribed to decrease pressure and swelling in the normal tissue around the tumors. For more details, see Table 1. This study was approved by the Ethical Committee of Iran University of Medical Sciences (Ethics approval number: IR.IUMS.FMD.REC.1397.085). Before entering the study, all participants were verbally informed of the aim and signed the consent form freely, according to the Declaration of Helsinki. All tissue samples were immediately placed into RNAlater Stabilization Solution (SigmaAldrich; Merck KGaA, Darmstadt, Germany) and were kept at -80°C until more process. 

*DNA extraction*

Genomic DNA was extracted from all fresh tissue samples (N=106) by using the QIAamp DNA Mini Kit according to the manufacturer’s instructions (Qiagen, Germany). The quality and quantity of isolated DNA were specified with a Nanodrop spectrophotometer (Thermo Fisher Scientific, USA) at the end of the extraction procedure. The extracted DNA can be stored at −20° prior to performing the polymerase chain reaction (PCR) reactions, if necessary.

*Polymerase chain reaction*

The presence of the EBV genome was detected by PCR with specific primers for *EBNA3C* and *EBNA-2* genes (Ai et al., 2012; Kato et al., 2013). Briefly, The reaction mixture contained containing 100 ng to 1 μg of template DNA, Taq DNA Polymerase 2x Master Mix (Amplicon, Denmark), 10 nM of each primer, and water up to 25 μl and PCR assay was carried out by Mas-tercycler personal (Eppendorf, Hamburg, Germany) under the following program: one cycle of initial denaturation at 95°C for 5 min (*EBNA3C*), 3 min (*EBNA-2*) and; 35 cycles of denaturation at 95°C 45 s(*EBNA3C*), 15s (*EBNA-2*); hybridization at 56°C for 45s (*EBNA3C*), 63°C for15s (*EBNA-2*), extension at 72°C for 1min (EBNA3C), the 30s *(EBNA-2*), and finally elongation at 72°C for 10 min (*EBNA3C*), 1 min (*EBNA-2*).

*Real-time quantitative PCR*

The quantification of *miR122* and *miR-BART1-3p* expression levels were conducted by SYBR green quantitative PCR (qPCR) kit (Qiagen, Germany). Briefly, total RNAs were isolated from CNS tumors tissues and healthy control tissues by TRIzol reagent (Invitrogen Life Technologies, Carlsbad, CA, USA), and PCR was performed by miScript SYBR Green PCR Kit (Qiagen, Valencia, CA; #218073), according to the manufacturer’s instructions. The qRT-PCR was performed in triplicate on an ABI PRISM 7000 (Applied Biosystems). The thermocycler conditions were as follows: 95°C for 2 minutes, followed by 40 cycles of 94°C for 15 seconds, 60°C for 20 seconds, and 70°C for 25 seconds. The ΔΔCt values were utilized to 2^-∆∆CT^ method and U6 small nuclear RNA (as an internal control) were used to analysis of relative gene expression data.

*Statistical analysis*

Continuous data were displayed as mean ± standard deviation or median ± IQR for normal and non-normal distribution, respectively. The comparison of the study groups was made through two sample t-test or Mann– Whitney U-test, while categorical data were displayed as N (%) and compared using *χ*^2^ or Fisher’s exact test. To measure the miRNAs expression, the Kruskal–Wallis H-test was used. However, correlation analysis was done by Spearman’s correlation coefficient between the *miRNA* expression and *CD4, NEF*, viral load and age. Moreover, false discovery rate was corrected by Benjamini–Hochberg approach for multiple comparisons. All statistical analyses were performed in STATA 14.2 (TX USA) and GraphPad Prism version 6 (CA, USA). Any p-value less than 0.05.

**Figure 1:**
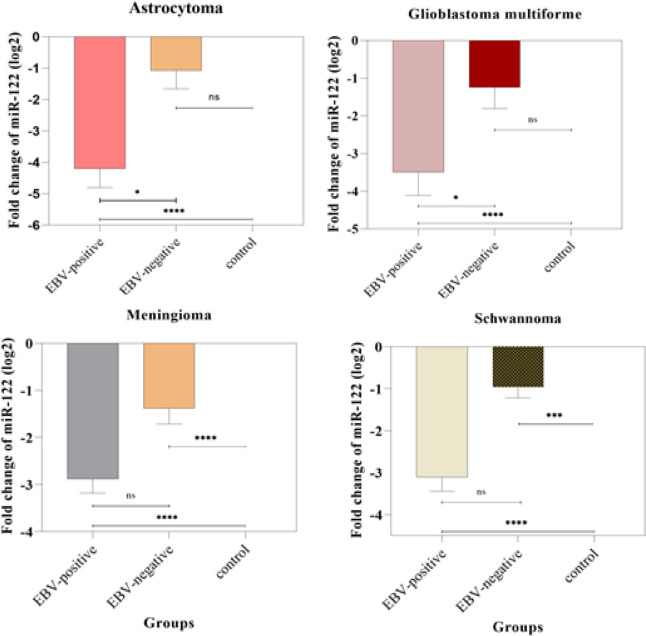
Comparison of the miR-122 Expression in Astrocytoma, Glioblastoma Multiforme, Meningioma and Schwannoma Tissue Samples. In astrocytoma and glioblastoma multiforme samples, the expression level of miR-122 in the EBV-positive group was significantly different from the EBV-negative group (*, P ≤ 0.05). The expression level of miR-122 was significantly downregulated in EBV-positive astrocytoma, glioblastoma multiforme, meningioma, and schwannoma, compared to the control group (**, P ≤ 0.01; ****, P ≤ 0.0001).

**Table 1:** Characteristics of Cases and Controls

Histopathology	Number (%) N=106	sex (M: F) (55:51)	Mean age (range)	Mean WBC counts per microliter (range)
Schwannoma	15 (14.1%)	9:06	45.7 (25-56)	8151.4 (4500-10950)
Meningioma	13 (12.2%)	8:05	63.1 (49-83)	9067.5 (6190-14150)
Glioblastoma multiform	12 (11.3%)	3:09	55.1 (20-74)	6838.5 (3400-9100)
Astrocytoma	9 (8.5%)	4:05	48.9 (18-67)	9596.6 (6800-11900)
Pituitary adenoma	(8.5%)9	6:03	54.3 (46-65)	8948.3 (7650-10450)
Epidermoid tumour	7 (6.6%)	5:02	39.2 (33-44)	11436.6 (6210-15490)
Adenocarcinoma (metastatic)	7 (6.6%)	4:03	47.7 (39-60)	8960.0 (5400-16700)
Hemangioblastoma	7 (6.6%)	1:06	51.6 (48-54)	7985.0 (6620-8750)
Pineoblastoma	6 (5.7%)	3:03	12.9(11-16)	7591.0 (6100-9300)
Oligodendroglioma	6 (5.7%)	4:02	44.1 (25-56)	8051.4 (4500-10950)
Oligoastrocytoma	5 (4.7%)	2:03	60.1 (49-83)	8567.5 (6190-14150)
Chordoma	5 (4.7%)	2:03	56.9 (20-74)	6838.5 (3400-9100)
Squamous cell carcinoma (metastatic)	5 (4.7%)	4:01	51.3 (18-67)	9090.0 (6800-11900)

**Figure 2:**
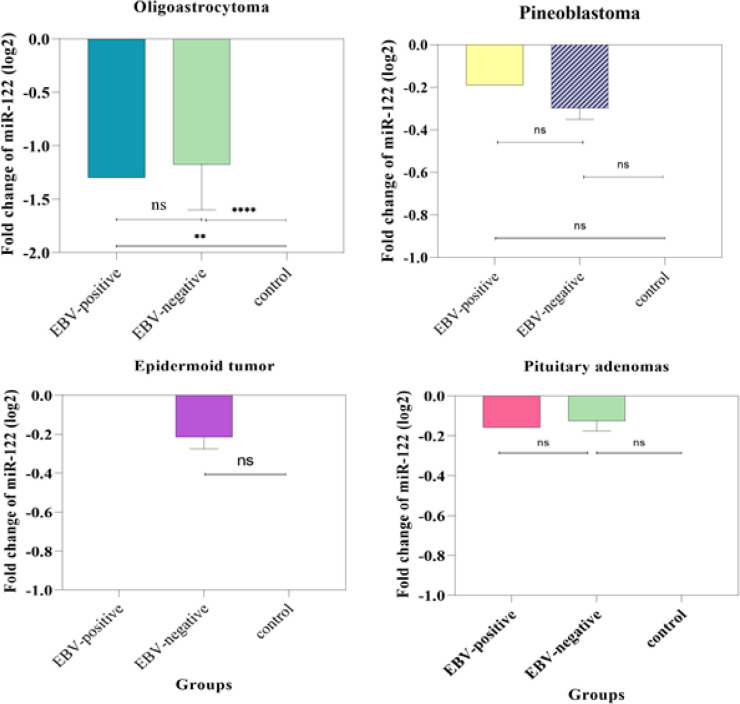
Comparison of the miR-122 Expression in Oligoastrocytoma, Pineoblastoma, Epidermoid Tumour, and Pituitary Adenomas Tissue Samples. The expression level of miR-122 was significantly downregulated in EBV-positive and EBV-negative oligoastrocytoma compared to the control group but, there is no significant difference in the expression of miR-122 between the EBV-negative and EBV-positive groups (**, P ≤ 0.01; ****, P ≤ 0.0001). The expression level of miR-122 was not significantly different between groups in pineoblastoma, epidermoid tumour, and pituitary adenomas tissue samples (ns, P > 0.05).

**Table 2:** Age, Sex, Cause of Death for Non-Neurological Disease Controls (n=32)

Cause of death	Age	Sex (Male & Female)
Coronary Artery Thrombosis	40	M
Abdominal Injuries	61	M
Asthma	52	F
Multiple Injuries	50	M
Pulmonary Thromboembolism	54	M
Bronchopneumonia	39	M
Atherosclerotic Cardiovascular Disease	49	F
Acute Myocardial Infarction	51	F
Hypertensive Arteriosclerotic Cardiovascular Disease	61	M
Congestive Heart Failure	49	M
Sickle Cell Disease	18	F
Sudden Cardiac Death	31	F
Breast Cancer	70	F
Asthma	15	M
P. aeruginosa Sepsis	19	F
Hypertensive Arteriosclerotic Cardiovascular Disease	49	M
Abdominal Injuries	72	F
Hypertensive Arteriosclerotic Cardiovascular Disease	76	M
Atherosclerotic Cardiovascular Disease	35	F
Hypertensive Arteriosclerotic Cardiovascular Disease	52	F
Atherosclerotic Cardiovascular Disease	61	F
Abdominal Injuries	33	M
Asthma	55	M
Abdominal Injuries	63	F
Hypertensive Arteriosclerotic Cardiovascular Disease	80	F
Atherosclerotic Cardiovascular Disease	48	M
Acute Myocardial Infarction	36	F
Hypertensive Arteriosclerotic Cardiovascular Disease	60	M
Acute Myocardial Infarction	67	F
Abdominal Injuries	43	M
Atherosclerotic Cardiovascular Disease	72	F
Hypertensive Arteriosclerotic Cardiovascular Disease	48	F

**Table 3:** Comparison of the Characteristics between EBV Positive and EBV Negative Cancer Tissues

		EBV Positive	EV Negative	Total	P-value
Number		18 (17%)	88 (83%)	106	
Age (years)		47±8 (20-83)	45.0 ± 17.3 (11-47)	46 ±17 (11-83)	0.56
WBC counts per microliter		8947.3 ± 3301 (4920-16700)	8102.3 ± 2310.5 (3390-15490)	8514.8 ± 2461.5 (3610-16700)	0.155
Tumour Location	Meninge	3 (2.84%)	15 (14.15%)	18	0.11
	Brain	15 (14.15%)	73 (68.86%)	88	
Gender	Male	8 (7.54 %)	47 (44.33%)	55	1
	Female	10 (9.43%)	41 (38.67%)	51	
Tumour Pathology	Schwannoma	3 (20%)	12 (80%)	15 (14.15%)	0.452
	Meningioma	3 (23%)	10 (77%)	13 (12.26%)	
	Glioblastoma multiform	4 (33.3)	8 (66.7%)	12 (11.32%)	
	Astrocytoma	4 (44.4%)	5 (55.6%)	9 (8.5%)	
	Pituitary adenomas	1 (11%)	8 (89%)	9 (8.5%)	
	Epidermoid tumour	1 (14.28%)	6 (85.7%)	7 (6.6%)	
	Adenocarcinoma (metastatic)	-	7 (100%)	7 (6.6%)	
	Hemangioblastoma	-	7 (100%)	7 (6.6%)	
	Pineoblastoma	1 (16.6%)	5 (83.4%%)	6 (5.7%)	
	Oligodendroglioma	-	6 (100%)	6 (5.7%)	
	Oligoastrocytoma	1 (20%)	4 (80%)	5 (4.7%)	
	Chordoma	-	5 (100%)	5 (4.7%)	
	Squamous cell carcinoma (metastatic)	-	5 (100%)	5 (4.7%)	

**Table 4:** Comparison of the miR-122 Expression between CNS Tumour Tissues and Healthy Controls

Tumour type	Fold change *-	log2	Expression level	P-value
Control	1	0	Unchanged	NA
Astrocytoma	0.18	-2.47±0.67	Down	<0/0001
Glioblastoma multiforme	0.25	-2±0.51	Down	<0/0001
Meningioma	0.3	-1.73±0.31	Down	<0/0001
Schwannoma	0.38	-1.39±0.31	Down	0/0001
Oligoastrocytoma	0.41	-1.2±0.68	Down	0.001
Pineoblastoma	0.82	-0.28±0.14	Down	ns
Epidermoid tumour	0.88	-0.18±0.15	Down	ns
Pituitary adenomas	0.91	-0.13±0.13	Down	ns

**Table 5:** Comparison of the miR-BART 1-3p Expression between EBV Positive Tissues and Healthy Controls

Expression level	EBV+ cancer tissue (N = 18)	EBV+ Control tissue (N=2)	Fold Change	P-Value	Adjusted P- value
miR-BART1-3p	15.36 ± 0.35	7.15 ± 5.37	2.14	<0.01	0.001

**Table 6:** Prevalence of EBV in Brain Tumours

Author	Country	Sample (Number)	Method	Detection result	Ref
Neves et al. 2008	Portugal	Tissue formalinfixed Pilocytic astrocytoma (n=35)	Real time PCR	9 (26%)	32
Cimino et al.2014	USA	Tissue formalinfixed Highgrade glioma (n=21)	NGS	5 (24%) Glioblastoma	33
Fonseca et al. 2015	Brazil	Fresh tissue: astrocytoma grade1 (n=7), astrocytoma grade2 (n=25), astrocytoma grade3 (n=16), GBM (n=18), ependymoma (n=5), oligodendroglioma (n=2), oligoastrocytoma (n=2) Total (n=75)	PCR	11 (14.7%) Astrocytoma G2 (6), Astrocytoma G3 (2), GBM (1), Oligoastrocytoma (1), Ependymoma (1)	28
Lin et al.2016	USA	Tissue formalinfixed Glioblastoma multiform (n=19)	Multiplex droplet digital PCR	4 (21%)	27
Strojnik et al. 2017	Slovenia	Fresh tissue glioma Grade (34) 45	Real time PCR	3 (6.7%) Glioblastoma	8
ZavalaVega et al. 2017	Mexico	Tissue formalinfixed Glioblastoma multiform (n=21)	Immunohistochemistry In situ hybridization	6 (28.6%)	30
Limam et al. 2019	Tunisia	Tissue formalinfixed: pilocytic astrocytoma (n=12), glioma grade2 (n=6), glioma grade3 (n=6), GBM (n=82), Total (n=112)	PCR	24 (21.4%) GBM	34
Khoury et al.2013	USA	Fresh tissue: Glioblastoma (n=168), Lowegrade glioma (n=47), Total (215)	RNA Sequencing	None positive	35
Cosset et al. 2014	Switzerland	Fresh tissue and serum Glioblastoma multiform (n=20)	Semiquantitative PCR	None positive	36
Hashida et al. 2015	Japan	Tissue formalinfixed Glioblastoma multiform (n=35)	Real time PCR	None positive	37
Sanjiv et al. 2015	Canada	Tissue formalinfixed Vestibular Schwannoma (n=121)	Immunohistochemistry PCR	None positive	38

## Results

A total of 106 cases (55 males and 51 females) and 32 healthy controls (male 15, female 17) were enrolled in our study which 87.7% (93/106) of samples were primary CNS tumors and the rest were metastatic CNS tumors. The mean age in the case group and control group were 46± 17.6 (mean ± standard deviation) and 47.42± 13.29 years, respectively. Of the four tumors with CNS metastasis, seven cases were diagnosed as adenocarcinoma (originating from lung cancer) and five cases were diagnosed as squamous cell carcinoma with the unknown primary site of origin. The primary CNS tumors samples in this study were categorized according to histopathologic criteria as follows: schwannoma (15/93, 14.1 %), meningioma (13/93, 12.2 %), and glioblastoma multiform (12/93, 11.3 %). Histologically, astrocytoma, pituitary adenoma, epidermoid tumors, hemangioblastoma, pineoblastoma, oligodendroglioma, oligoastrocytoma, and chordoma, respectively, were the less common tumors. The mean of the WBC count was 8438.8 ± 2645.5 cells per microliter (range, 3400-16700 WBC per microliter). More info1*323rmation was shown in Table 1.

EBV DNA was detected in 17% (18/106) of tumors tissue samples and 6.4% (2/32) of control tissues using the PCR method. In EBV positive tumors tissues, the most prevalence was in Astrocytoma (44.4%) and Glioblastoma multiform (33.3%) had the highest prevalence of the EBV DNA. According to the results, statistically significant correlation was found between the presence of EBV and CNS tumors (p=0.001). In addition, the association between histopathological types of CNS tumors and EBV was not statistically significant (R=0.61). More details are presented in Table 3.

In the Table IV, Figure 1 and 2, the expression level of *miR-122* was compared between CNS tumors tissues and healthy controls, also between EBV positive and EBV negative tissues. according to results, the expression level of *miR-122* was upregulated in squamous cell carcinoma, Chordoma, Adenocarcinoma tissue samples compared to control samples, but the increase in expression was not significant (Table 4). Also, the expression level of *miR-122 *was significantly downregulated in astrocytoma, glioblastoma multiforme, meningioma, schwannoma, oligoastrocytoma, and hemangioblastoma tumors tissue samples than in normal tissues (Figures 1 and 2, p<0.05, Table 4). Besides, the expression level of *miR-122* was not significantly different between groups in pineoblastoma, epidermoid tumors, and pituitary adenomas tissue samples (Figure 2, ns. p>0.05). Moreover, the results showed that the miR-122 was differentially expressed in histopathological type of CNS tumors (Table 4). Also, among all the samples, the highest reduction in the expression level of *miR-122 *was observed at astrocytoma tumors samples (fold change = -2.47±0.67). However, the correlations between the expression of *miR-122* and types of CNS tumors were significantly positive (0.035). More information is shown in Table IV. 

The expression level of the *EBV miR-BART1-3p* was significantly upregulated in the EBV–positive tumors tissues compared to EBV–positive control tissues (p<0.01) (Table 5).

## Discussion

Brain tumors include 2% of all tumors in patients and glioma and meningioma are the most frequent types, however, its reason is yet unknown and needs further research (Bergo et al., 2016; Langen et al., 2017; Zavala-Vega et al., 2017a). Various molecular biology-based techniques indicate a link between viral infections and brain tumors but is not still clear which virus plays a major impact on brain tumorsigenesis and progression (Strojnik et al., 2017; Fonseca et al., 2015; Leibovitch et al., 2016; Zavala-Vega et al., 2017a; Akhtar et al., 2018b). In 1964, EBV was identified as the first human oncovirus which is one of the important risk factors in human cancers originating from lymphocytes, epithelial cells, and mesenchymal cells (Moghoofei et al., 2019; Ko, 2015). In this study, we indicated that EBV infected 17.24% of tumors tissue samples and 6.4% of control tissues. According to the results, among the EBV-infected tumors samples, EBV-DNA had the highest prevalence in astrocytoma (4/9, 44.4%), and glioblastoma multiforme (4/12, 33.3%), respectively and in general, a statistically significant relationship was found between the presence of EBV and CNS tumors (p < 0.001). Leibovitch et al. investigated the relationship between some herpes viruses (EBV, HCVM, and HHV-6A, B) and astrocytoma in 112 brain tissue specimens by using PCR (PCR) method. Although the HCMV and HHV-6A genomes were not detected in any of the samples, HHV-6B and EBV were detected in 13.3% and 8.9% of the astrocytoma samples, respectively. Only EBV was statistically positive in that study (Leibovitch et al., 2016). In the present study, the EBV genome was found in 44.4% of tissue samples of patients with astrocytoma. 

Zavala-Vega et al., (2017b) investigated the rate of EBV, CMV and HSV 1/2 infection in the brain tissue of GBM patients by using in situ hybridization (ISH), real-time PCR, and immunohistochemistry (IHC). The mono-infection of CMV, HSV-1/2, and EBV were detected in 4.8%, 19%, and 28.6%, respectively. Furthermore, co-infection of CMV- EBV, HSV-1/2- EBV and also HSV-1/2-EBV- CMV were detected in 23.8%, 19%, and 4.34%, respectively. In the current study, we detected the EBV-DNA genome in 33.3% of GBM tissue samples. Furthermore, Cosset et al., (2014) investigated the presence of VZV, EBV, HHV-6, HSV, CMV in 20 brain tumors biopsies. They reported that none of those viruses were detected in meningiomas, low-grade gliomas, oligoastrocytoma, oligodendroglioma, and ependymoma. 

Unlike, in the current study, the EBV genome was found in meningioma (3/13, 23%) oligoastrocytoma (1/5, 20%) samples and similarly, the virus genome was not detected in any sample of oligodendroglioma tissue (Table 3).

In the study performed by Poltermann et al. (Poltermann et al., 2006) reported that IgG antibodies against EBV in the serum of 8.33 of patients with acoustic schwannomas and also, in our study, EBV was detected by PCR method in 20% tissue samples of patients with schwannomas. These differences may be due to the methods used to detect the virus (Further studies are shown in Table 6).

The first human virus known to encode viral-miRNAs was the EBV virus that encoding miR-BART (miR-BART 1 to miR-BART 22) and miR-BHRF1 (miR-BHRF1 to miR-BHRF3) (Barth et al., 2011). Several studies shown that EBV actively uses its own miRNAs to regulate cellular and viral pathways during oncogenic transformation (Barth et al., 2011). Some studies have reported that EBV oncoproteins were not expressed in nasopharyngeal carcinoma (NPC) infected with EBV, so it may be possible that other mechanisms contribute to tumors progression. However, Chen et al., (2010) reported that three EBV-miRNAs including miR-BART9, 5 and 3 were overexpressed in all NPC tissues examined. In addition, EBV-BART9 leads to the upregulation of LMP1 mRNA levels and increases growth rates in Nasal NK T Cell Lymphomas (Ramakrishnan et al., 2011). In another study performed by Hsu et al., (2014) have been demonstrated which miR-BART9 through inhibiting of E-cadherin promotes the invasiveness and migration of NPC cells. For the first time, Ye et al., (2013) investigated gene expression profiles among metabolism-associated genes in NPC and EBV-miR-BART1 in by the RNA deep sequencing approach. They found that this miRNA is associated with the metabolism of NPC and maybe play critical roles in NPC development. It has been observed that miR-BART1 is upregulated in NPC cells and EBV-miR-BART1 causes promote tumors metastasis by regulating PTEN-dependent pathways in NPC. However, the role and expression level of miR-BART1 in brain tumors has not been well studied. In this study, we assessed the miR-BART1-3p levels in EBV-positive tissue samples and results demonstrated that miR-BART1-3p level significantly increased in EBV positive tumors samples compared with the EBV-positive control samples (p<0.01). Also, this study is the first report of the expression level of miR-BART1-3p in human CNS tumors tissues. 

Nowadays, because of the importance of miRNAs as a biomarker in the diagnosis of various disorders, including cancer and viral diseases, has received much attention (Nahand et al., 2019). Dysregulation of miRNAs is associated with different pathological cellular conditions such as cancer, viral infection, inflammation and etc. (Witwer, 2015). Thus, they can be used as important diagnostic biomarkers. For example, miR122 has been reported to be associated with heart diseases and suggested that probably overexpression of this miRNA implicated in the proliferation and apoptosis of cardiomyocytes (Zhang and Jing, 2018). As well, it has reported which increased circulating miR-122 acts as a discrimination biomarker between sepsis and infection (Rahmel et al., 2018) and has potential as a new, predictive, and reliable marker for chemical, alcohol-, and viral-induced liver injury (Zhang et al., 2010). In addition, the miR-122 levels has been investigated in various cancer cell types and observed that acts as both a tumors suppressor in hepatocellular carcinoma (Zeisel et al., 2012), gastric cancer (Rao et al., 2017) breast cancer (Wang et al., 2012) and as an oncomiR (miRNA that is associated with cancer) in renal cell carcinoma (Jingushi et al., 2017). However, the function of this miRNA as oncomiR or tumors suppressor in CNS/brain cells is not yet fully understood. In the study conducted by the Wang and colleagues the miR-122 level was assessed in the normal group and glioma patients. They demonstrated that the level of this miRNA was decreased in glioma patients compared to healthy subjects (Wang et al., 2014). In another study, Tang et al indicated that the miR-122 levels in the serum of glioma patients downregulated and levels of miR-122 could be used as a prognostic factor (Tang et al., 2017). In the current study, a statistically significant difference between the levels of miR-122 in different tumors types (P=0.0001) was observed. Also, the expression level of miR-122 was downregulated in EBV positive tumors samples compare to the EBV negative samples (P<0.001). Notably that the greatest reduction in the expression level of miR-122 was in that of astrocytoma and GBM which these samples had the highest prevalence of EBV. It has been found that viruses by deregulation of miRNAs expression can alter cellular function to their advantage. In general, these results suggest that the miR-122 may act as a suppressor tumors in CNS and that the EBV infection can contribute to the progression of the tumors by dysregulation of* miR-122* expression.

In summary, we demonstrated that the prevalence of EBV in CNS tumors was 17% and statistically, there is a significant relationship between the presence of EBV-DNA with CNS tumors. Also, the level of* EBV-BART1-3p* expression was significantly higher in EBV-positive tumors samples than EBV-positive normal samples. Besides, the expression level of miR-122 was significantly decreased in the EBV-positive sample compared to that of the EBV-negative sample. In general, the results of this study suggest that the EBV could change the condition of cancer cells by altering the expression of miR-122 and EBV-BART1-3p and maybe contribute to the development of cancer cells. These findings highlights the importance of the further investigations in this field.

## Data Availability

All data generated or analyzed during this study are included in this published article.
